# Comparing integrated training of the hand and arm with isolated training of the same effectors in persons with stroke using haptically rendered virtual environments, a randomized clinical trial

**DOI:** 10.1186/1743-0003-11-126

**Published:** 2014-08-23

**Authors:** Gerard G Fluet, Alma S Merians, Qinyin Qiu, Amy Davidow, Sergei V Adamovich

**Affiliations:** Department of Rehabilitation and Movement Science, Rutgers The State University of New Jersey, Room 714C, 65 Bergen Street, Newark, NJ 07101 USA; Department of Quantitative Methods, Rutgers University, 65 Bergen Street, Newark, NJ USA; Department of Biomedical Engineering, New Jersey Institute of Technology, University Heights, 65 Bergen Street, Newark, NJ USA

**Keywords:** Stroke, Upper extremity, Hand, Arm, Virtual reality, Robotics

## Abstract

**Background:**

Robotically facilitated therapeutic activities, performed in virtual environments have emerged as one approach to upper extremity rehabilitation after stroke. Body function level improvements have been demonstrated for robotically facilitated training of the arm. A smaller group of studies have demonstrated modest activity level improvements by training the hand or by integrated training of the hand and arm. The purpose of this study was to compare a training program of complex hand and finger tasks without arm movement paired with a separate set of reaching activities performed without hand movement, to training the entire upper extremity simultaneously, utilizing integrated activities.

**Methods:**

Forty individuals with chronic stroke recruited in the community, participated in a randomized, blinded, controlled trial of two interventions. Subjects were required to have residual hand function for inclusion. The first, hand and arm separate (HAS) training (n = 21), included activities controlled by finger movement only, and activities controlled by arm movement only, the second, hand and arm together (HAT) training (n = 20) used simulations controlled by a simultaneous use of arm and fingers.

**Results:**

No adverse reactions occurred. The entire sample demonstrated mean improvements in Wolf Motor Function Test scores (21%) and Jebsen Test of Hand Function scores (15%), with large effect sizes (partial r^2^ = .81 and r^2^ = .67, respectively). There were no differences in improvement between HAS and HAT training immediately after the study. Subjects in the HAT group retained Wolf Motor Function Test gains better than in the HAS group measured three months after the therapy but the size of this interaction effect was small (partial r^2^ = .17).

**Conclusions:**

Short term changes in upper extremity motor function were comparable when training the upper extremity with integrated activities or a balanced program of isolated activities. Further study of the retention period is indicated.

**Trial registration:**

NCT01072461.

**Electronic supplementary material:**

The online version of this article (doi:10.1186/1743-0003-11-126) contains supplementary material, which is available to authorized users.

## Background

Sensorimotor impairments and participation restrictions remain a pervasive problem for patients post stroke, with recovery of upper extremity function particularly recalcitrant to intervention[1]. Repetitive task practice (RTP), the repetitive performance of goal oriented activities designed to develop more normal upper extremity movement patterns[2–4], has been used as a rehabilitation approach to redevelop the complex integrated control associated with normal upper extremity function in persons after stroke[2].

Many researchers are developing robotic-assisted arm training devices to facilitate the delivery of RTP. Robotic systems offer several advantages in the ability to deliver RTP, including the ability to provide highly repetitive practice with systematic increases in task difficulty, graded assistance as well as the ability to turn small active movements into goal directed activities[5, 6]. Most robotic therapies have focused on isolated training of the proximal effectors of the upper extremity. Two systematic reviews that examined the effects of isolated robotic training of the proximal effectors found moderate improvements in proximal motor impairments as measured by the Fugl-Meyer Assessment of Upper Extremity Function (UEFMA) but no statistically significant improvements at the activity or participation level[5, 6]. A few groups have examined robotically facilitated interventions targeting the hand for persons with strokes[7–10]. In small trials, this training approach has produced moderate improvements in proximal as well as distal motor function and subjects in two of these trials demonstrated improvements at the activity and participation level as well[11].

Virtual reality based training systems offer many of the proposed benefits of robot-based therapies without the haptic feedback or assistance[12]. In multiple studies, persons with stroke have demonstrated improvements at the motor function level in response to programs of VR based proximal UE training[13]. Persons with stroke have also demonstrated improvements at the activity level using a VR based program of isolated hand training[14]. These studies suggest that there might be a need to train the hand to elicit activity and/or participation-level change.

A majority of the real-world studies on RTP in persons with stroke incorporate activities that integrate activity of the hand wrist and fingers. This level of integration may be necessary to produce activity and or participation level change due to the enormous complexity required for normal upper extremity activity. The isolated nature of a majority of the virtually simulated and robotically-aided rehabilitations studied to date may have limited the transfer of motor function improvements developed during these interventions into improvements in the ability of subjects to use their upper extremity (UE) to interact with objects in the real world.

Three robotic devices that allow integrated movements of the hand and arm have been tested[15–17]. Subjects performing integrated UE training in a study by Klamroth-Marganska et al.[15] demonstrated better improvements at the impairment level as measured by the UEFMA than the traditional therapy group. It is important to note that the differences in UEFMA changes demonstrated by both the integrated robotic training condition and the traditionally presented training condition was relatively small and that the changes in activity and participation level measures demonstrated by the two groups did not differ. This said, their results make a statement speaking to the effectiveness of integrated robotic UE training compared to usual care but do not establish an additive effect for integrated robotic training when compared to isolated robotic UE training.

Krebs et al. utilized a set of activities incorporating an isometric grasp component in addition to several mixed reality activities combining robotically facilitated reaching movements and grasping of real world objects to the MIT-MANUS system and compared them to training robotically facilitated reaching movements only, finding better proximal impairment level improvements for isolated training[16]. In a cross-over study using the robotic BONES exoskeleton, Milot et al. compared multi-joint functional robotic training with single joint, impairment-based training. Both approaches in the Milot study demonstrated similar impairment, activity and participation level improvements[17]. These two studies support the use of robotic training for impairment and functional based improvement post-stroke, but cast doubt on the need to train whole upper extremity complex movement patterns in order to achieve transfer to activities of daily living. However, it is important to note that neither of the isolated training paradigms included a targeted finger training component and in each of the studies the nature of the tasks presented in the two training conditions differed substantially as well.

The purpose of this study was to compare a training program of complex hand and finger tasks without arm movement paired with a separate set of reaching activities performed without hand movement, to training the arm and fingers simultaneously, utilizing integrated activities. Both programs were performed using the NJIT RAVR and TrackGlove systems[18, 19] and a suite of simulated activities provided similar sensorimotor challenges and comparable feedback. It is believed that the UE operates in an integrated fashion during most real-world functional movements thus appearing to support the concept that task specific training using whole arm movements may be more beneficial than isolated joint training. Thus, we hypothesized that a VR/robotic system that simultaneously trained all the joints of the UE would have a greater impact on real-world functional movement than a system that trained proximal and distal limb segments separately.

## Methods

This study is registered at http://clinicaltrials.gov/show/NCT01072461 and was approved by the institutional review boards of the New Jersey Institute of Technology and Rutgers University.

### Participants

**Figure 1:**
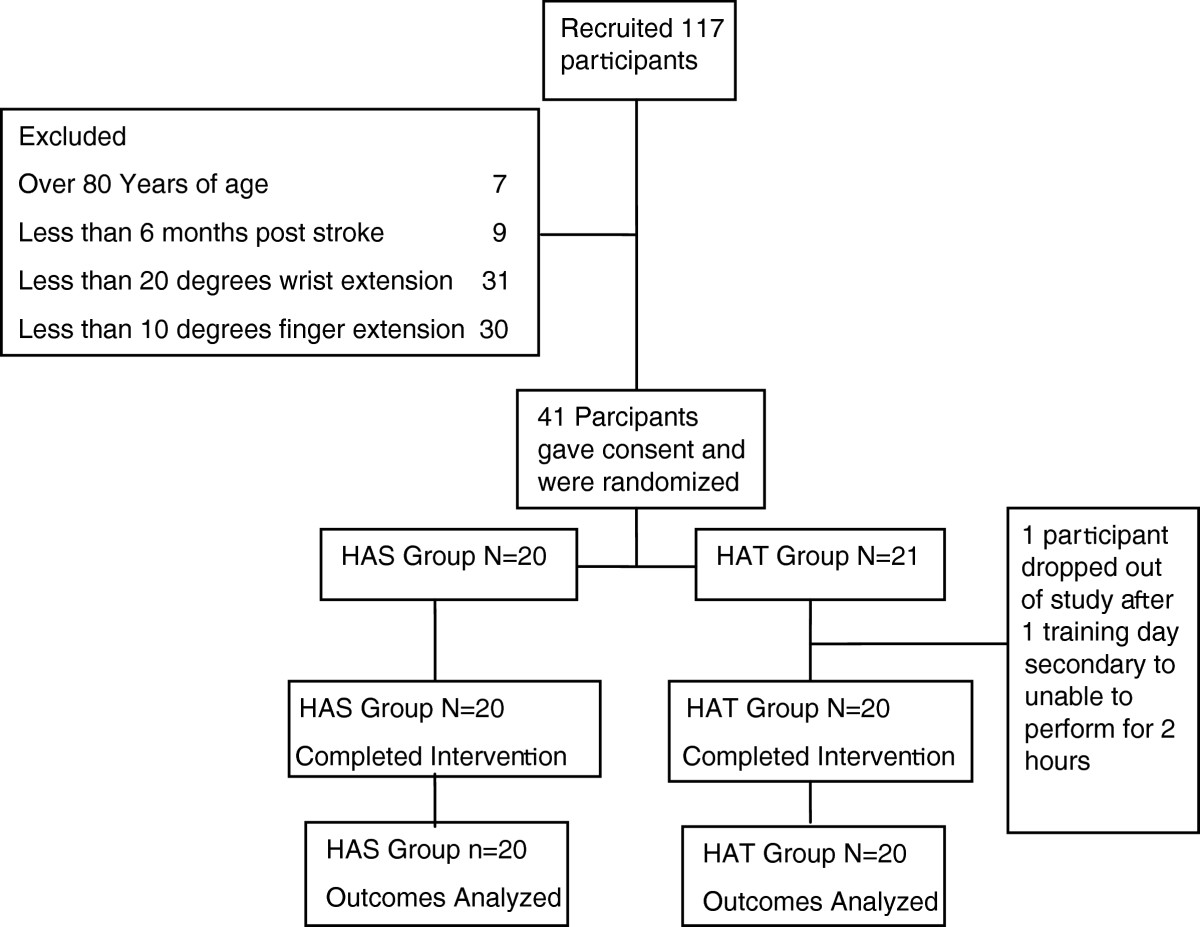
**CONSORT diagram.** Describes participant flow through screening, randomization, data collection and intervention.

Potential subjects (Figure 1) were screened for participation using the following inclusion criteria: 1) between 18 and 80 years of age, 2) at least 6 months post-stroke, and 3) at least 20° of wrist extension and 10° of finger extension[18], and 4) able to flex shoulder and extend elbow without pain. Potential subjects were excluded if they had received botulinum toxin injections in the three months prior to recruitment, or if they demonstrated aphasia that rendered them unable to participate in the consent process, or sensory/perceptual issues that did not allow subjects to perform impaired hand movements without looking at their hand. Subjects needed to be able to follow three step commands and attend to task for an entire ten minute screening session. Informed consent was obtained from all subjects.

### Randomization

A person not involved in data collection performed group assignment, using a random number table. Odd numbered subjects were randomized to hand and arm separate training (HAS); even numbered subjects were randomized to hand and arm together training (HAT).

### Testing

Following randomization, subjects completed a battery of clinical tests two weeks prior to training and one day prior to training. A third testing session followed three days after completion of an eight day period of training and a follow-up testing session occurred three months after training. Testing was performed by a physical therapist, blinded to group allocation and the nature of the training programs. Subjects agreed to not participate in other therapy targeting their upper extremity during the training and testing period as a condition of participation in the study.

### Training systems

**Figure 2:**
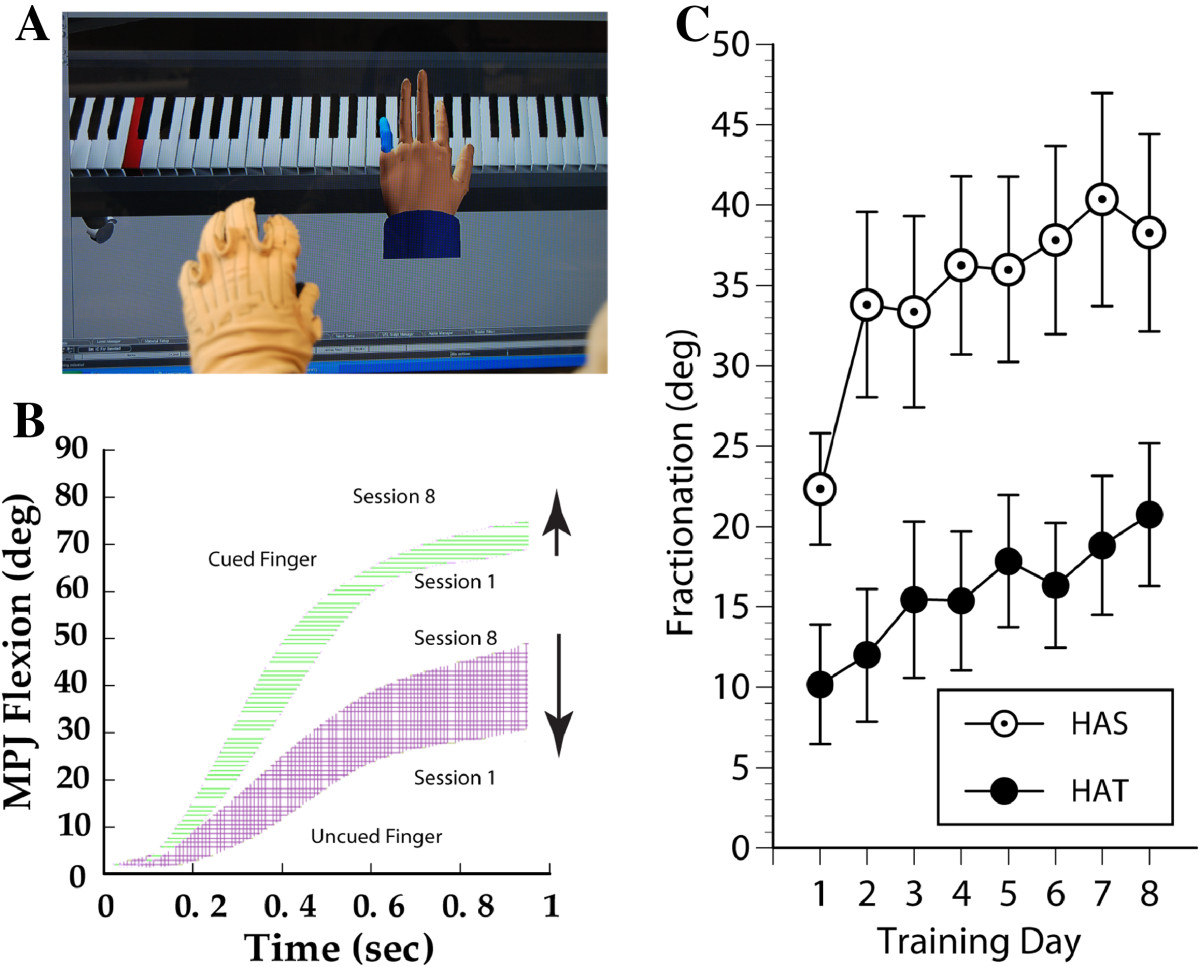
**Virtual piano trainer. A**: NJIT TrackGlove system, with the subject’s hand in the foreground and a screenshot of the Virtual Piano Trainer simulation in the background. **B**: Data for a single repetition of the Virtual Piano Trainer Simulation performed on Training Day 1 and another repetition on Training Day 8 performed by a representative subject. The horizontally hatched area represents the change in cued finger flexion secondary to training. Cued finger flexion angle increased slightly (top pair of lines). Non-cued finger flexion decreases more extensively (bottom set of lines) secondary to training, with the cross hatched area between the two lines indicating improved ability for finger individuation. **C**: Daily averages for finger fractionation score for HAS group subjects (open circles) and HAT group subjects (solid circles) during Virtual Piano Trainer simulation performance. Error bars represent the standard error of the mean. Please see text for further explanation of findings.

The NJIT-Track Glove system- consists of a CyberGlove™ (Immersion, USA), which is an instrumented glove for finger angle tracking, and a TrackStar™ three-dimensional magnetic tracking system (Ascension Technology, USA) used to track hand position and orientation. The glove acts as an interface between the participants and the virtual environments[18] (see Figure 2).

**Figure 3:**
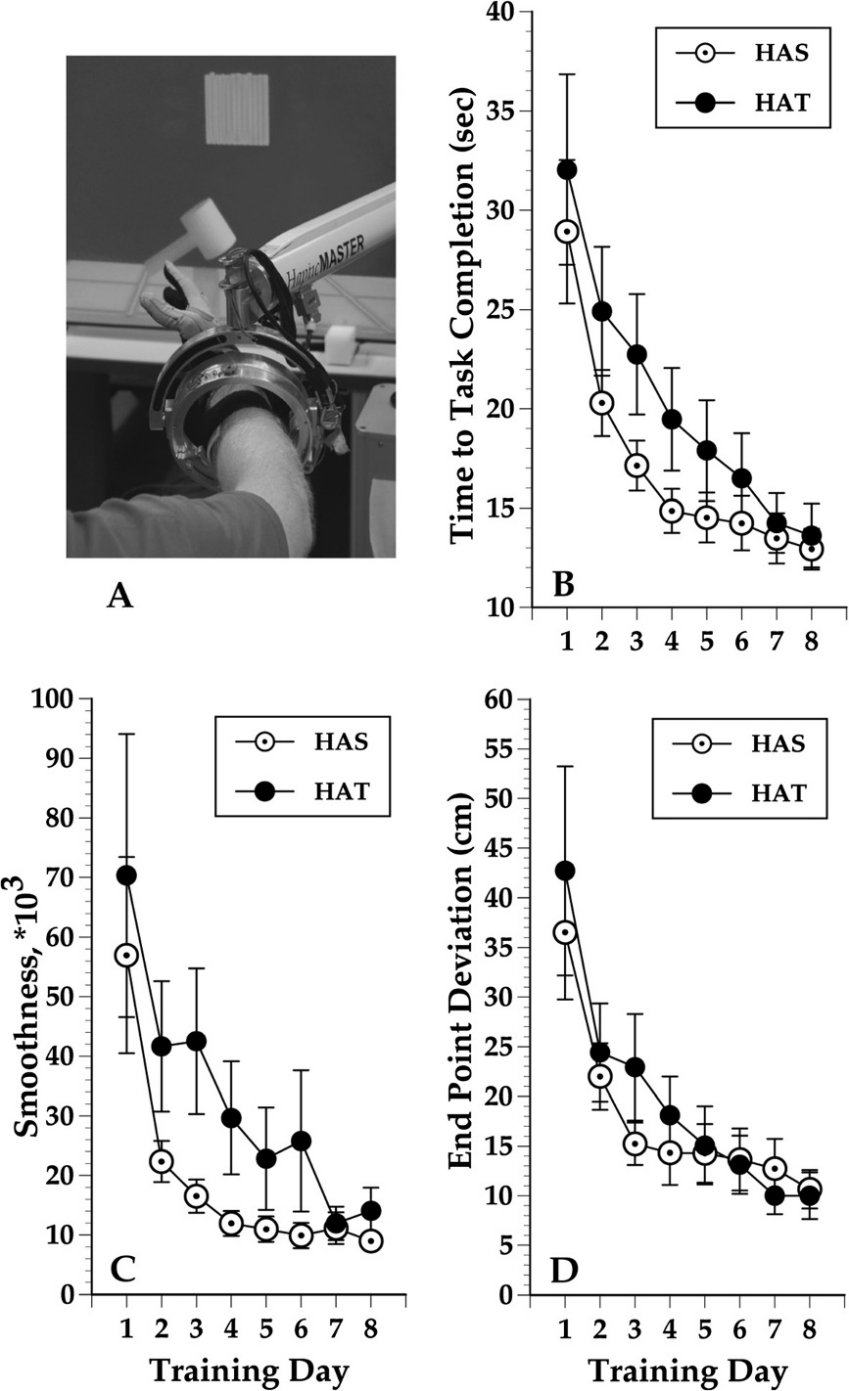
**Hammer Task Simulation. A**: NJIT RAVR System in foreground and a screenshot of the Hammer Task simulation in the background. **B-D**: Daily averages for Time to Task Completion **(B)**, Trajectory Smoothness **(C)** and End Point Deviation **(D)** for HAS group subjects (open circles) and HAT group subjects (solid circles) during Hammer Task simulation performance. Lower smoothness scores indicate better performance. Error bars represent the standard error of the mean. Please see text for further explanation of findings.

The NJIT RAVR system consists of the CyberGlove (described above), combined with the Haptic Master, a 6 degrees of freedom robot (Moog, The Netherlands). The robotic arm provides tracking of multi-planar movements against gravity in a 3D workspace and enables programmable haptic effects, such as variable anti-gravity support, springs and dampers, and haptic objects, such as walls, floors, tables and other complex-shaped objects[19] (see Figure 3).

### Training protocol

**Figure 4:**
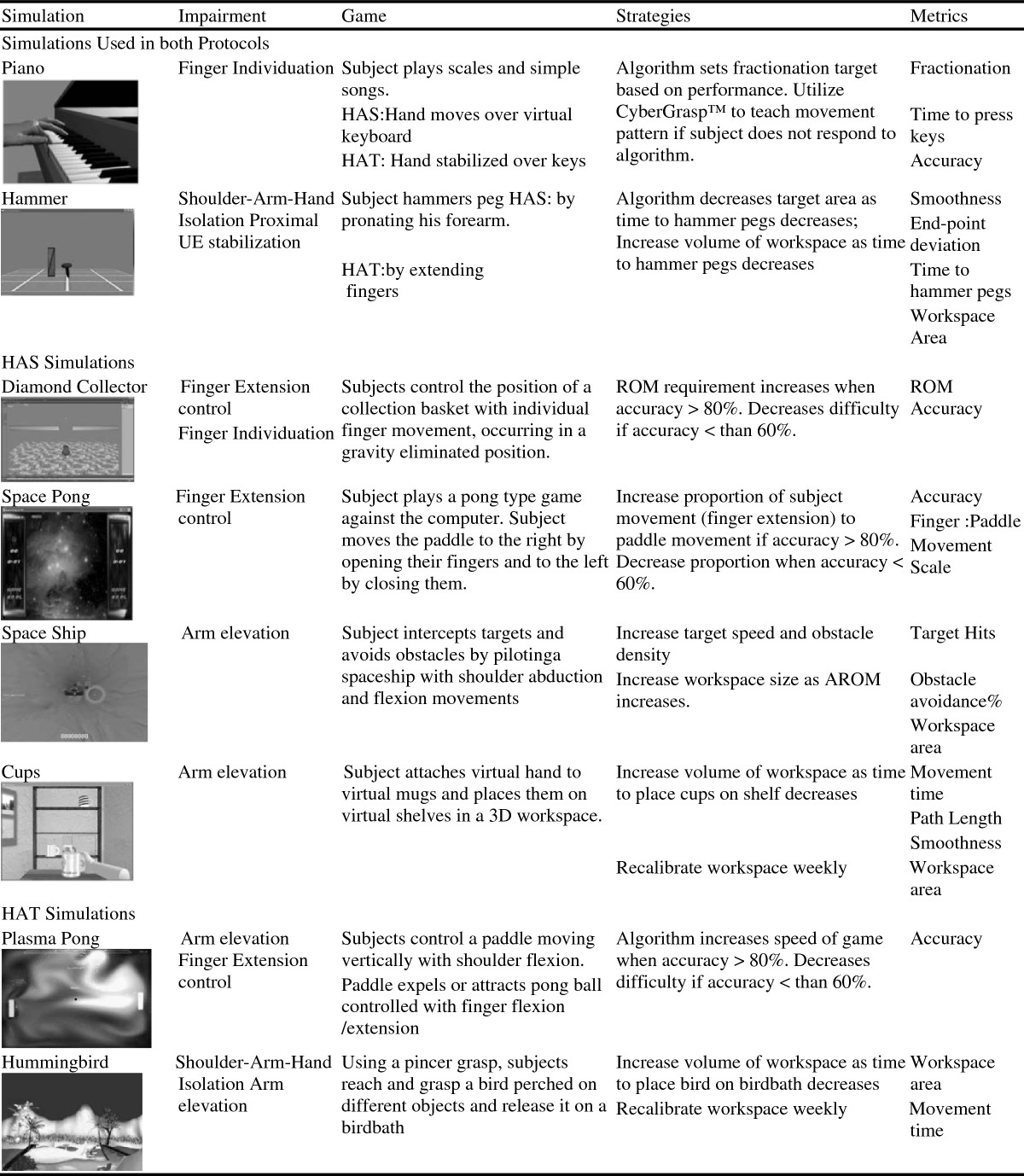
**Training simulations.**

The HAS group engaged in virtually simulated rehabilitation tasks for hand movements for half of each training session using the NJIT TrackGlove system, then performed movements for the shoulder and elbow only using the NJIT RAVR system for the other half of the session (see Figure 4). The HAT group trained the exact same amount of time using the NJIT TrackGlove and NJIT RAVR system but all of the simulations required integrated shoulder, elbow, wrist and hand use (see Figure 4). To standardize postural control across groups, during training all subjects were seated with seat height adjusted to maintain their feet flat on the floor and their femurs parallel to the ground. Seat depth was adjusted to insure adequate support of the femurs and contact between the trunk and back of the seat, without excessive posterior pelvic tilt. The work space of the training simulations that involved reaching utilized a calibration protocol that measured active upper extremity range of motion achieved without movement of the trunk away from the backrest of the seat. Training was performed in a workspace between sixty and eighty percent of the maximum reaching space achieved during calibration.

All subjects performed two hours of training on Day 1, and progressed to three hours by Day 4, which continued to Day 8. Subjects had a single five minute break as they moved between the NJIT TrackGlove and NJIT RAVR training stations. The only other breaks were for transitions between simulations which took less than 60 seconds. Subjects trained four consecutive days on week one and four consecutive days on week two. Several subjects rescheduled sessions within the two training weeks and two needed to extend training into a third week because of inclement weather. Two training weeks were chosen based on the success of the EXCITE trial. The two to three hour sessions and four day training weeks were chosen based on responses to training of subjects performing pilot testing completed during development of the training systems.

### Outcome measures

The primary outcome measure for this study was the Wolf Motor Function Test (WMFT) because of its balanced testing of the arm and hand as well as its use in a major upper extremity (UE) rehabilitation study of persons with stroke[20]. Secondary outcome measures included the Jebsen Test of Hand Function (JTHF) and kinematic measures collected during the training*.* Both the WMFT and the JTHF test at the activity and performance level of the International Classification of Function and Health[21]. Kinematic measures identify changes at the body function level.

The WMFT is a battery of fifteen timed tasks utilized to describe the ability of a person with stroke to move their shoulder, elbow and hand. The WMFT has high levels of inter-rater reliability (ICC ≥ 0.97) and test-retest reliability (ICC = .90)[22] and is able to discriminate between the impaired and unimpaired upper extremities of persons with stroke[23]. Elapsed time was recorded for each of the 15 items. A score of 120 seconds was recorded if subjects were unable to perform an item. A sum of the fifteen elapsed times, including times of 120 seconds for items that were not completed, was recorded as a subject’s score. This differs from other applications of the WMFT but is consistent with our previously published studies. We used this approach to account for changes in motor ability demonstrated when subjects could perform items at post or retention testing that they could not perform at pre-test. This approach resulted in fifteen item scores for all subjects, for all measurement periods.

The modified JTHF is a battery of six standardized tasks that times the ability of the hand and fingers to manipulate and transport small objects. It has good levels of intra-rater reliability (r = .72) and good concurrent validity with the Action Research Arm Test (ARAT) (r = .87) and Nine Hole Peg Test (r = .84) in persons with stroke[24]. Elapsed time was reported for each of the six items[25]. If a subject was unable to perform an item in 45 seconds, the task was terminated and this time was recorded as their score[26]. All *s* ubjects performed the WMFT and JTHF in a seated position, following the positioning guidelines described in the training protocol section.

Kinematic measures of shoulder and elbow function during performance of the Hammer-Task simulation (time to task completion, end-point trajectory smoothness and end-point deviation during hammering) and kinematic measures of hand/finger function during Virtual Piano performance (finger fractionation, a measure of finger individuation) were recorded during the eight training days. These two simulations were chosen because they are discrete tasks. Scores reported for each measure were averages of every repetition performed on each training day.

Three kinematic measurements were collected during hammer task training. 1) Time to task completion, which is the average time to complete reaching for and hammering a target peg. This demonstrates the ability to reach efficiently and produce repetitive distal movements while stabilizing the proximal extremity. 2) Trajectory smoothness, which numerically describes the ability to produce smooth, coordinated, gross reaching movements and may be an indicator of neurological recovery in persons with strokes[27]. In a previous study subjects demonstrated positive correlations between improvements in trajectory smoothness and improvements in motor function[26]. 3) End point deviation is a measure of the ability of the shoulder and elbow musculature to stabilize the entire upper extremity during the object interaction portion of the hammer simulation[28]. The score is calculated as the mean excursion of the avatar from the target peg during the hammering motion (finger flexion/extension or pronation/supination).

One kinematic measure was collected during virtual piano trainer performance, fractionation, which measures the ability of the participant to flex a cued finger to a greater extent than non-cued fingers. It measures both the ability of the single cued finger to flex actively outside of a mass flexion pattern and the ability of the remaining non-cued fingers to remain stable. This stabilization requires a combination of activation of the non-cued finger extensors as well as de-activation of the non-cued finger flexors[18], see also[28] for a detailed discussion of this concept. Each of these abilities is associated with neurologic recovery and improved hand function in persons with stroke[29]. FS is calculated as the angle of the active finger’s metacarpo-phalangeal joint minus the MCP angle of the most flexed inactive finger[18]. When the active finger flexes beyond the most flexed inactive finger the value is positive. When the inactive fingers are flexed beyond the active finger, the value is negative.

### Data analysis

For the primary outcome measures, we hypothesized that both posttest and retention scores would differ by treatment group, after controlling for each of the individual pre-test scores. We tested our hypothesis with two separate linear regression models (one for post test scores and another for retention scores), using estimation via maximum likelihood methods[30]. Because we tested posttest and retention scores separately, the value of 0.025 was used to declare statistical significance based on Bonferroni criterion. Partial r-squared was used to estimate the effect size, identifying the proportion of the variability in the scores that was due to the treatment group, after controlling for pre-test scores. Because posttest and retention test scores were not linearly related to pre-test scores, the pre-test scores were treated as 4 level categorical variables; ≤40 seconds (pre-test1: 4 subjects and pre-test 2: 6 subjects), 40-80 seconds (10 subjects and 15 subjects) , 80-120 seconds (4 subjects and 10 subjects) and > 120 seconds (11 subjects and 9 subjects)[31]. These categories were assigned based upon the distribution of pre-test WMFT scores with a goal of at least 4 subjects per category (approximately 10%) which is typical for this approach to modeling[31]. For all estimated regression models, residuals were normally distributed, eliminating the need for the log normalization typically associated with WMFT scores.

Eleven subjects (7 HAT, 4 HAS), performed only one clinical pre-test because of scheduling issues. To evaluate the impact of these missing data on hypothesis testing, we repeated all statistical analyses controlling only for the single pre-test measurement collected immediately prior to training for each subject. The results of these statistical analyses did not differ from the results of the statistical analyses that included all the available pre-test measurements. We will report only the results of comparisons using two pretests for subjects who completed both pre-tests and a single pretest score for subjects that completed only one. All subjects completed post-test measurement. A single HAS subject did not perform retention testing due to a secondary stroke.

For the primary outcome measures, JTHF scores were analyzed similarly to the WMFT scores, except that the pre-test scores were categorized into 3 levels; <90 seconds (pre-test1: 9 subjects and pre-test 2: 9 subjects), 90-150 seconds (9 subjects and 16 subjects) and, > 150 seconds (11 subjects and 9 subjects). Kinematic measures obtained during training exercises Piano and Hammer (fractionation, time to task completion, smoothness and end point deviation) on Day 1 were compared with those collected on Day 8 using the Wilcoxon rank-sum test. Day one training values varied between the two groups due to differences in the HAS and HAT versions of the simulated training tasks (see Figure 4). To assess whether treatment group had an effect on improvement in kinematic measures over the course of the intervention, we estimated regression models of Day 8 values which included the main effects of Day 1 values and treatment group, plus terms capturing the interaction between Day 1 values and group. Interaction terms were used to test whether a group effect depended upon the pre-test values. As in the WMFT and JTHF analysis, each kinematic variable’s Day 1 values were categorized into 4 levels due to non-linearity considerations[31].

Six subjects’ (2 HAS, 4 HAT) fractionation scores from the Piano exercise were not included in the statistical analyses because they had to use a hand exoskeleton[18] to assist them during this activity. Finally, 4 subjects’ scores from the Hammer exercise (3 HAS, 1 HAT) were not included in the statistical analyses because the activity was substantially modified (fixation of the arm above the target was provided by the robot during hammering) to allow for their participation. All of these subjects performed other simulations in the same fashion as the rest of their cohort and followed the same training schedule.

## Results

Forty-one subjects were randomized into the two treatment conditions (please see CONSORT Figure 1 for results of the recruitment and screening process. There were no significant differences in the patient characteristics or severity of stroke, as measured by the Chedoke McMaster Impairment Inventory, between the groups (Table 1)[32]. None of the subjects experienced an adverse response to treatment. On training day one, HAS group participants averaged 1579 (±1083) repetitions while HAT group participants performed an average of 1146 (±1117) repetitions. On training day eight, HAS group participants averaged 2585 (±1335) and HAT group research participants averaged of 2414 (±9607) repetitions. There was no statistically significant difference between HAS and HAT groups in the total repetitions performed on any single day or for the entire course of the intervention.

**Table 1:** **Subjects characteristics by group**

	HAT group N = 20	HAS group N = 20	T-test/FET/WRST
Age, mean (CI),	56.0 (49.5-62.4)	53.1 (48.4-57.9)	t_1,38_ = .73, p = .47
Gender, M/F, n	15/5	14/6	FET, p = .50
Pre-Morbid Handedness, Right/Left, n	16/4	19/1	FET, p = .17
Affected UE, Right/Left, n	10/10	13/7	FET, p = .26
Time since onset, median (IQR), mo	41.5 (85.5)	48.5 (.5)	WRST, p = .91
CMA stage (max = 6), median (IQR)	5.0 (1.0)	5.0 (1.7)	WRST, p = .55
CMH stage (max = 6), median (IQR)	5.0 (1.0)	4.0 (2.5)	WRST, p = .64

*CI* = 95 percent confidence interval, *FET*: Fischer’s Exact test, *WRST*: Wilcoxon rank-sum test, *IQR* – Interquartile range, *CMA*: Chedoke McMaster Arm Impairment Stage[38], *CMH*: Chedoke McMaster Hand Impairment Stage[38].

### WMFT changes (pre to post-test)

Overall improvement in WMFT from pre-test to post-training for all forty subjects was statistically significant. On average, WMFT scores were 26.2 seconds lower than values obtained at pre-test (95% CI = [14.8, 37.7], p < 0.0001, with a large effect size of partial r^2^ = 0.81). For the 29 subjects with two pre-test scores collected, WMFT scores did not vary significantly from pre-test one to pre-test two (paired t-test; p = 0.15). Subjects in the HAS group demonstrated a mean improvement from pre-test to post-test of 30.8 seconds. This change was larger than the 21.6 second improvement demonstrated by the HAT group. However, the post-test WMFT score was not statistically different across the two groups of subjects: p = 0.41, controlling for both pre-test measurements (Table 2).

**Table 2:** **Clinical and kinematic measurement changes**

Test/Measure	Group	Pre-Test/Day 1	Post-Test/Day 8	Retention	Pre-post change	Pre-retention change
WMFT, mean (CI), sec	HAS N = 20	117.2 (77.5-156.9)	86.4 (60.7-112.1)	91.7 (64.5-118.8)	30.8 (12.6 – 49.0)	21.8 (-1.0-44.7)
	HAT N = 20	92.4 (60.8-124.1)	70.8 (45.8-95.7)	62.5 (44.9-80.1)	21.6 (5.5-37.8)	29.9 (9.31-50.5)
JTHF, mean (CI), sec	HAS N = 20	146.8 (120.4-173.2)	126.4 (103.6-149.2)	131.6 (107.2-156.1)	20.4 (7.5 – 33.3)	14.4 (-0.04-28.8)
	HAT N = 20	124.8 (100.9-148.7)	107.1 (83.5-130.6)	113.7 (90.5-137.0)	17.7 (8.5-27.0)	11.1 (1.1-21.0)
Finger Fractionation, median (IQR), deg	HAS N = 18	21.7 (12.4-35.4)	40.1 (17.3-59.6)		14.8 (6.11-26.8)	
	HAT N = 16	10.9 (1.8-20.8)	24.5 (5.4-34.7)		8.6 (4.1-17.1)	
Time To Task Completion, median (IQR), sec	HAS N = 20	25.0 (20.6-36.4	12.7 (10.8-14.9)		9.9 (6.2-23.0)	
	HAT N = 20	25.4 (18.9-42.0)	14.7 (8.0-19.3)		12.5 (7.6-22.5)	
Reaching Trajectory Smoothness*, median (IQR)	HAS N = 16	28.1 (20.1-66.0)	8.2 (4.3-10.8)		19.2 (9.1-56.6)	
	HAT N = 20	35.7 (18.2-76.3)	8.6 (2.4-23.1)		23.3 (15.0-45.9)	
Endpoint Deviation, median (IQR), cm	HAS N = 16	27.1 (15.8-55.6)	9.1 (5.5-12.4)		16.3. (7.2-45.7)	
	HAT N = 20	38.0 (13.1-51.1)	6.2 (3.5-10.3)		25.6 (5.3-35.2)	

*CI* = 95 percent confidence interval, *IQR* = Interquartile Range, * = Reported as actual smoothness multiplied by 1,000.

### WMFT changes (pre-test to retention)

Overall, WMFT scores at retention were 25.8 seconds lower than at pre-test (95% CI = [11.6, 40.4], p =0.0003, partial r^2^ = 0.68). Subjects in the HAS group demonstrated a mean improvement from pre-test to retention of 21.8 seconds. This change was smaller than the 29.9 second improvement demonstrated by the HAT group. This time, the retention WMFT score was statistically different across the two groups of subjects: p = 0.0114, controlling for both pre-test measurements. However, the size of this effect was small, with the partial r^2^ associated with the group effect equal to 0.17, i.e., only 17% of the variability in the retention WMFT can be explained by the group, after controlling for the pre-test WMFT scores (Table 2).

### JTHF changes (pre to post-test)

Overall changes in JTHF for all forty subjects from pre-test to post-training were statistically significant (Table 2). On average, JTHF scores were 19.1 seconds lower than values obtained at pre-test (95% CI = [11.7, 26.4], p < 0.0001, with a large effect size of r^2^ = 0.67). Subjects in the HAS group demonstrated a mean improvement from pre-test to post-test of 20.4 seconds and for the HAT group, the improvement was 17.7 seconds. Controlling for both pre-test measurements, the p-value for the group effect was 0.40 (Table 2).

### JTHF changes (pre-test to retention)

Overall JTHF scores at retention were 12.7 seconds lower than at pre-test (95% CI = [4.5, 20.7], p <0.0001, with a large effect size of partial r^2^ = 0.78). HAS group demonstrated a mean improvement from pre-test to retention of 14.4 seconds. This change was not significantly different from the 11.1 second improvement demonstrated by the HAT group. Controlling for both pre-test measurements, the p value for the group effect was 0.39 (Table 2).

### Training kinematics

Overall, subjects demonstrated statistically significant improvements (p < 0.01) for all four kinematic variables as measured by the Wilcoxon rank-sum test (p = 0.009 for fractionation; all others p < 0.0001) (Table 2). Fractionation score in the Piano simulation improved from a median of 15.6 degrees on day 1 to 25.8 on day 8 (Figure 2). The three variables in the Hammer Task improved as well from day 1 values: time-to-task completion’s median was 25.0 sec on day 1 and 13.1 sec on day 8; smoothness’s median was 33,836.4 on day 1 and 8,403.9 on day 8; end point deviation had a median of 30.5 cm on day 1 and 7.6 cm on day 8 (Figure 3). Day one scores for fractionation differed between groups significantly. This can be explained by exacerbation of the UE flexion synergy occurring due to shoulder abductor loading, as subjects needed to move and stabilize the hand during the HAT version of the activity while the upper extremity was externally stabilized during the HAS version of the activity. It is important to note that both groups improve in a comparable pattern and that the interaction between measurement time and training group was not statistically significant. See also the large changes demonstrated between Day 1 and Day 2. These changes are usually associated with task familiarization and for many subjects, the sensorimotor transformations associated with performing a simulated activity. Figure 2 shows the change in fractionation for each group as well as a representational subject’s increased ability to stabilize the un-cued fingers in a less flexed position, resulting in an improved fractionation score.There were no statistically significant differences between groups for day one scores for any of the Hammer Task kinematic measurements (Figure 3). Figure 3 shows the changes in Hammer Kinematics for each group. Regression models that included day 1 values and the main effect of the treatment group (HAS vs. HAT), did not identify a statistically significant difference when comparing the improvements demonstrated by either group for any kinematic variable (fractionation: p = 0.50, arm fixation: p = 0.12, smoothness: p = 0.87, time to completion: p = 0.38). Note the differences in smoothness and time to completion early in the trial for the two versions of the task. This may be due to interactions between the hand and shoulder for the HAT version of the task. By the end of training scores for the two groups are essentially equal. This may be due to a ceiling effect related to the HAS version of this activity.

## Discussion

Both groups demonstrated improvements in the study’s primary outcome, the WMFT, as well as the JTHF and kinematic parameters acquired during the training. There were no differences between subjects that simultaneously trained all the joints of the UE and those that trained proximal and distal limb segments separately when measured immediately after training. However, interestingly, WMFT scores for subjects in the group that simultaneously trained all the joints of the UE, improved from posttest to retention test while WMFT scores for subjects in the isolated training group regressed slightly towards baseline scores. The similarity in the findings in pre-test to post-test outcomes between the two training programs in this study was contrary to our original hypothesis but similar to other studies that have addressed the issue of integrated UE robotic training in persons with stroke[16, 17].

If the biomechanical similarity between HAT training and UE function in the real world were the critical factor for the transfer of training benefits to our outcome measures, HAT training should have produced superior results, based on the concept of specificity of training. However, one of the hallmarks of motor learning is the ability to transfer improvements in motor performance to dissimilar tasks[33]. This said, several other factors associated with enhanced motor learning may be just as important as task specificity. Factors such as volume of training and type of feedback were controlled for in the design of these two training programs. This study’s results may suggest that the similarities in these other factors, discussed below, may have resulted in comparable levels of more general motor learning and therefore similar levels of transfer, to a broad range of dissimilar UE tasks.

There is substantial evidence supporting the hypothesis that the volume of rehabilitation activity has a significant impact on motor learning in general[34] and more specifically, the outcome of rehabilitation interventions in persons with stroke[35]. The workloads (in terms of time) performed by HAS and HAT group participants were comparable in this study. Difficulty of training is another factor proposed to have an impact on motor learning[34] as well as the outcome of rehabilitation interventions[35]. The HAT and HAS versions of the Hammer and Virtual Piano simulations both utilized algorithms to adjust the difficulty of tasks based on the success of the immediately preceding repetitions. We utilized workspace-scaling following a similar schedule for the balance of the simulations in the two programs. It could be argued that the equivalence of the two training programs in terms of difficulty of training effectively controlled for any possible confounding effects of task complexity.

Newer research on the effects of simple feedback in persons with stroke, underscore the impact of feedback on motor adaptations subsequent to motor interventions[36]. The amount and types of feedback presented to both groups may have had an effect on the outcomes. Approximately forty percent of the two interventions presented identical feedback due to the Hammer task and Virtual Piano simulations being included in both treatment programs. Knowledge of results feedback was provided in the form of a game score for one of the HAT simulations (Plasma Pong) and two of the HAS simulations, one for proximal effectors (Space-Ship) and one for distal effectors (Space Pong). While the feedback was not identical across the two training approaches, the factors described in this paragraph would support that feedback across the training conditions was roughly equivalent and that this equivalence is consistent with the comparable outcomes observed.

Differences in the retention patterns of our primary outcome measure across the two groups of subjects were significant although the effect size was small. Explanation of these differences and confirmation of their clinical relevance will require further study. The two training protocols may have elicited different changes at the substrate level with the adaptations made by subjects who trained all degrees of freedom of the UE simultaneously (including finger flexion/extension) proving to be more durable than those made by subjects in the isolated training group. Alternatively, the two training patterns may have transferred to different patterns of behavior following training that resulted in continuing improvement in the use of the arm in the HAT training subjects and a slight regression towards baseline in the HAS training subjects. We plan to add testing of neurophysiological adaptations to this type of training along with a more detailed study of activity levels during the retention period in our future investigations.

The mean percent improvement in WMFT score across all 40 subjects was 24.8% and the effect size was .81 when measured immediately after therapy. This large improvement in WMFT score demonstrated by both groups in this study when compared to other studies of technology based upper extremity rehabilitation in persons with stroke that train the proximal upper extremity only[20, 37, 38] support the notion that training the hand along with the proximal upper extremity is critical for transfer of the training to interactions with objects in the real world. Further investigation into differences in the retention of this transfer of training will be necessary to determine if integrated whole upper extremity training should be chosen over programs of isolated activities for the arm and isolated activities of the hand. Another issue related to generalization of our results and a definitive answer to our research question is the moderate to minimally impaired levels of motor function in our sample. Overall response to VR/Robotic training and the pattern of similar adaptation to the two types of training may prove different in a sample of subjects with lower levels of motor function.

## Conclusion

This study failed to identify a significant difference in activity level improvements elicited by integrated, robotically facilitated UE training and a comparable dose of isolated, robotically facilitated UE training in a group of persons with mild to moderate hemiparesis due to chronic CVA. The findings of this study have potential implications on the design of upper extremity rehabilitation systems. Robotic and/or virtually simulated activities integrating large excursion movements of the proximal upper extremity with smaller excursion, but more complex movements of the wrist and fingers are more challenging to design and implement than activities training these two sets of effectors separately. Complex, robotic systems integrating several joints are also more costly and more time consuming to set up, making them difficult to incorporate into clinical practice when compared to simpler robotic equipment that trains a single effector[11]. Further investigation into the retention effect should clarify whether additional health-care dollars spent on integrated training systems versus simpler isolated training systems are warranted.

## Authors’ original submitted files for images

Below are the links to the authors’ original submitted files for images. Authors’ original file for figure 1 Authors’ original file for figure 2 Authors’ original file for figure 3 Authors’ original file for figure 4
