# Supplementary figures and images for: Comparing integrated training of the hand and arm with isolated training of the same effectors in persons with stroke using haptically rendered virtual environments, a randomized clinical trial
Source: J Neuroeng Rehabil. 2014 Aug 23;11:126. doi: 10.1186/1743-0003-11-126 (PMC4156644; doi:10.1186/1743-0003-11-126)

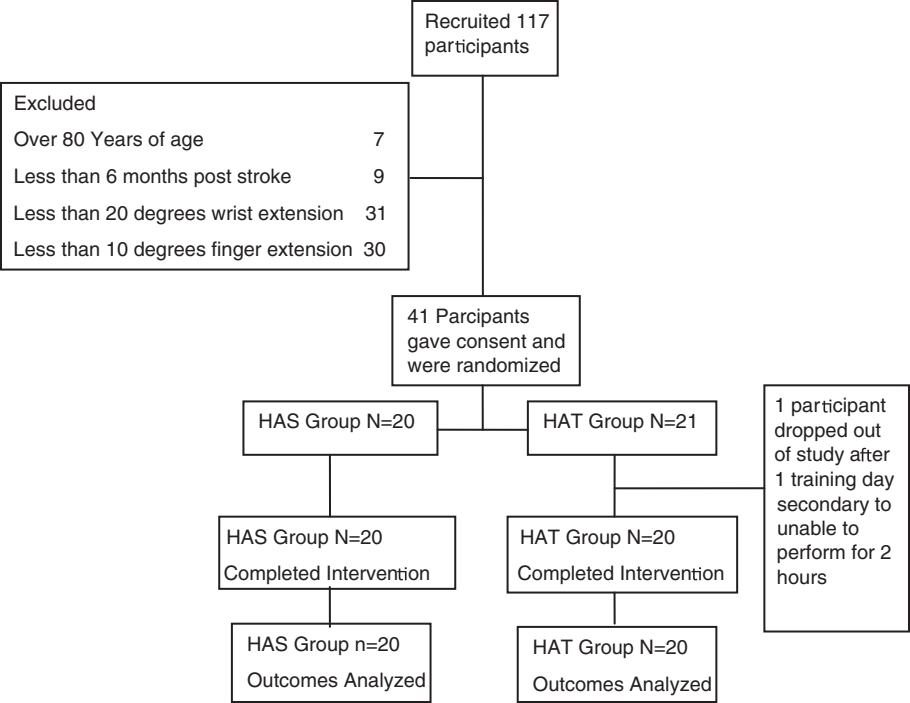

Supplement: Supplementary file 1 — Authors’ original file for figure 1 [file 12984_2014_649_MOESM1_ESM.pdf]

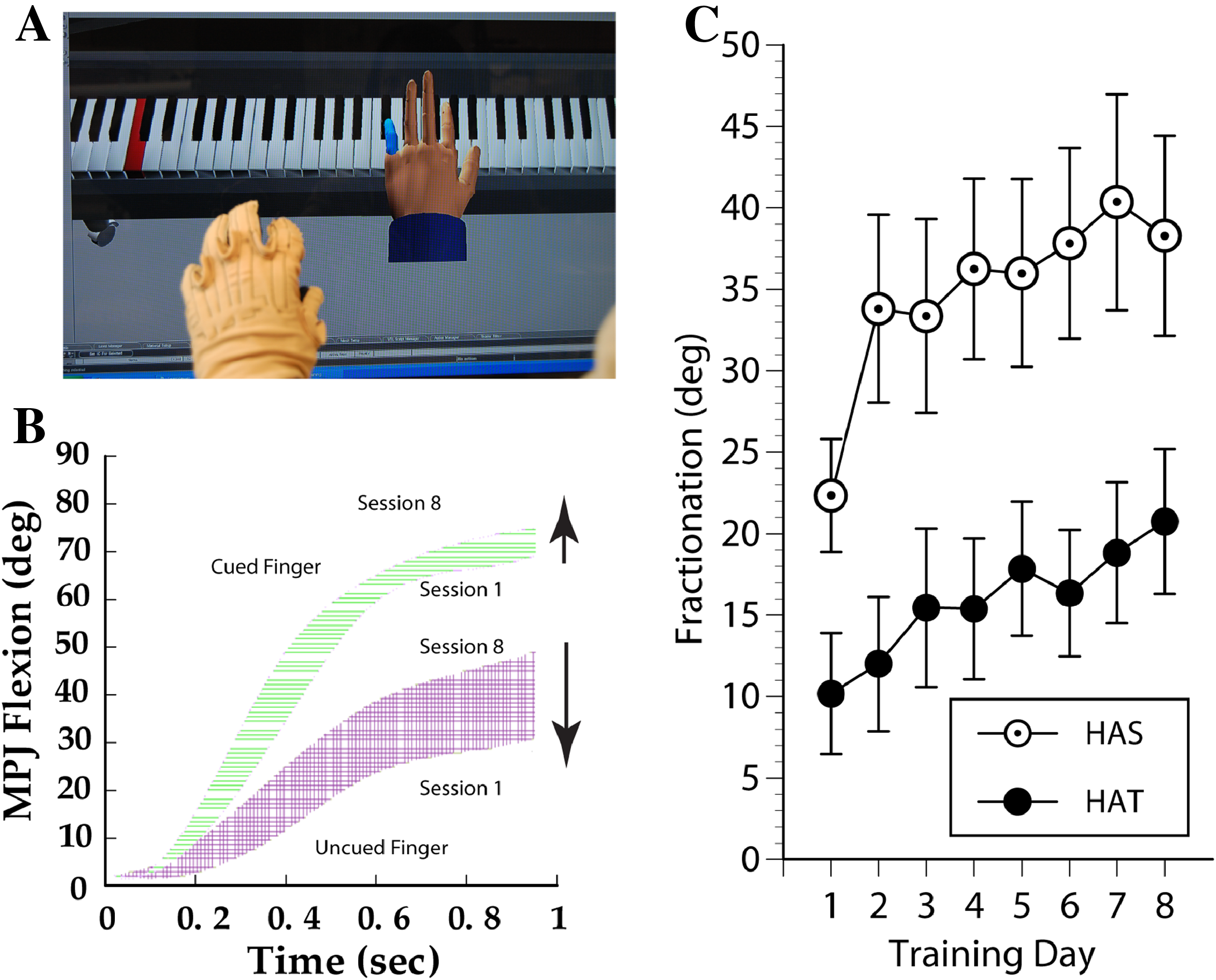

Supplement: Supplementary file 2 — Authors’ original file for figure 2 [file 12984_2014_649_MOESM2_ESM.tif]

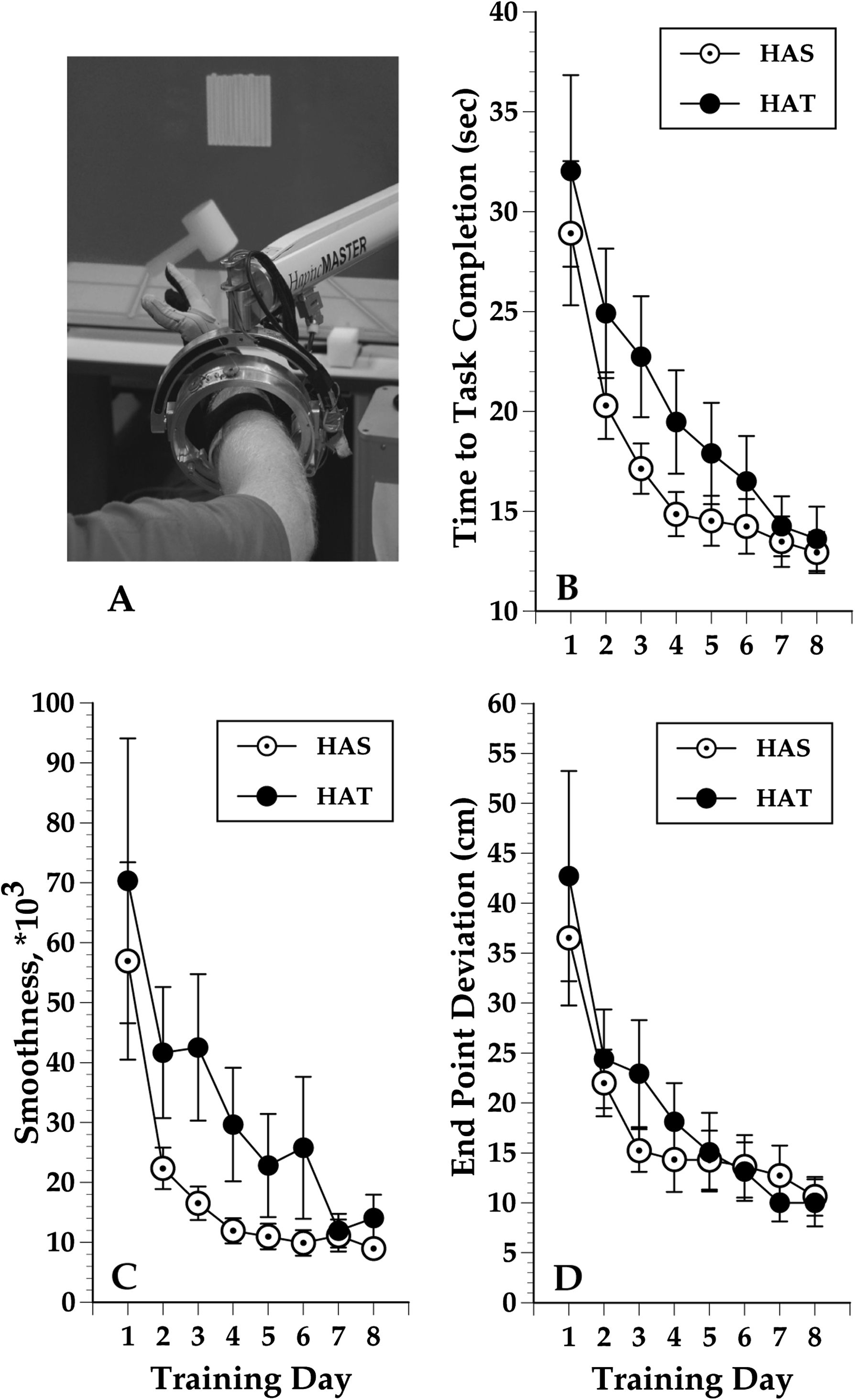

Supplement: Supplementary file 3 — Authors’ original file for figure 3 [file 12984_2014_649_MOESM3_ESM.tif]
