# Supplementary material for: Comparing integrated training of the hand and arm with isolated training of the same effectors in persons with stroke using haptically rendered virtual environments, a randomized clinical trial
Source: J Neuroeng Rehabil. 2014 Aug 23;11:126. doi: 10.1186/1743-0003-11-126 (PMC4156644; doi:10.1186/1743-0003-11-126)
Supplement: Supplementary file 4 — Authors’ original file for figure 4 [file 12984_2014_649_MOESM4_ESM.pdf]

Simulation	Impairment	Game	Strategies	Metrics
Simulations Used in both Protocols				
Piano<br>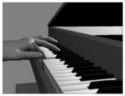	Finger Individuation	Subject plays scales and simple songs.<br>HAS:Hand moves over virtual keyboard<br>HAT: Hand stabilized over keys	Algorithm sets fractionation target based on performance. Utilize CyberGrasp™ to teach movement pattern if subject does not respond to algorithm.	Fractionation<br>Time to press keys<br>Accuracy
Hammer<br>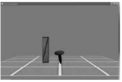	Shoulder-Arm-Hand Isolation Proximal UE stabilization	Subject hammers peg HAS: by pronating his forearm.<br>HAT:by extending fingers	Algorithm decreases target area as time to hammer pegs decreases; Increase volume of workspace as time to hammer pegs decreases	Smoothness<br>End-point deviation<br>Time to hammer pegs<br>Workspace Area
HAS Simulations				
Diamond Collector<br>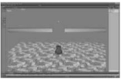	Finger Extension control<br>Finger Individuation	Subjects control the position of a collection basket with individual finger movement, occurring in a gravity eliminated position.	ROM requirement increases when accuracy > 80%. Decreases difficulty if accuracy < than 60%.	ROM<br>Accuracy
Space Pong<br>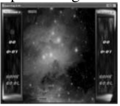	Finger Extension control	Subject plays a pong type game against the computer. Subject moves the paddle to the right by opening their fingers and to the left by closing them.	Increase proportion of subject movement (finger extension) to paddle movement if accuracy > 80%. Decrease proportion when accuracy < 60%.	Accuracy<br>Finger :Paddle Movement Scale
Space Ship<br>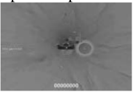	Arm elevation	Subject intercepts targets and avoids obstacles by pilotinga spaceship with shoulder abduction and flexion movements	Increase target speed and obstacle density<br>Increase workspace size as AROM increases.	Target Hits<br>Obstacle avoidance%<br>Workspace area
Cups<br>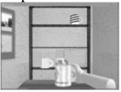	Arm elevation	Subject attaches virtual hand to virtual mugs and places them on virtual shelves in a 3D workspace.	Increase volume of workspace as time to place cups on shelf decreases<br>Recalibrate workspace weekly	Movement time<br>Path Length<br>Smoothness<br>Workspace area
HAT Simulations				
Plasma Pong<br>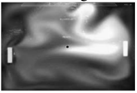	Arm elevation<br>Finger Extension control	Subjects control a paddle moving vertically with shoulder flexion.<br>Paddle expels or attracts pong ball controlled with finger flexion /extension	Algorithm increases speed of game when accuracy > 80%. Decreases difficulty if accuracy < than 60%.	Accuracy
Hummingbird<br>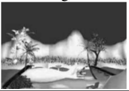	Shoulder-Arm-Hand Isolation Arm elevation	Using a pincer grasp, subjects reach and grasp a bird perched on different objects and release it on a birdbath	Increase volume of workspace as time to place bird on birdbath decreases<br>Recalibrate workspace weekly	Workspace area<br>Movement time
